# Temporal Contiguity Training Influences Behavioral and Neural Measures of Viewpoint Tolerance

**DOI:** 10.3389/fnhum.2018.00013

**Published:** 2018-01-30

**Authors:** Chayenne Van Meel, Hans P. Op de Beeck

**Affiliations:** Laboratory of Biological Psychology, Brain and Cognition, KU Leuven, Leuven, Belgium

**Keywords:** viewpoint tolerance, fMRI adaptation, temporal contiguity hypothesis, visual cortex, face perception

## Abstract

Humans can often recognize faces across viewpoints despite the large changes in low-level image properties a shift in viewpoint introduces. We present a behavioral and an fMRI adaptation experiment to investigate whether this viewpoint tolerance is reflected in the neural visual system and whether it can be manipulated through training. Participants saw training sequences of face images creating the appearance of a rotating head. Half of the sequences showed faces undergoing veridical changes in appearance across the rotation (non-morph condition). The other half were non-veridical: during rotation, the face simultaneously morphed into another face. This procedure should successfully associate frontal face views with side views of the same or a different identity, and, according to the temporal contiguity hypothesis, thus enhance viewpoint tolerance in the non-morph condition and/or break tolerance in the morph condition. Performance on the same/different task in the behavioral experiment (*N* = 20) was affected by training. There was a significant interaction between training (associated/not associated) and identity (same/different), mostly reflecting a higher confusion of different identities when they were associated during training. In the fMRI study (*N* = 20), fMRI adaptation effects were found for same-viewpoint images of untrained faces, but no adaptation for untrained faces was present across viewpoints. Only trained faces which were not morphed during training elicited a slight adaptation across viewpoints in face-selective regions. However, both in the behavioral and in the neural data the effects were small and weak from a statistical point of view. Overall, we conclude that the findings are not inconsistent with the proposal that temporal contiguity can influence viewpoint tolerance, with more evidence for tolerance when faces are not morphed during training.

## Introduction

The human visual system has a remarkable ability to reliably identify objects and faces across large variations in appearance. This skill requires a trade-off between selectivity – which allows us to differentiate between highly similar objects (e.g., two faces) – and tolerance – which is needed to recognize the same object across large changes in appearance (e.g., one face seen at different viewing distances, from different angles or under different lighting conditions). Selectivity and tolerance have both been shown to be affected by learning. Learning increases selectivity in human behavior (e.g., Goldstone, 1994, 1998; Folstein et al., 2012), in the human brain (e.g., Freedman et al., 2006; Gillebert et al., 2009; Folstein et al., 2013; Brants et al., 2016), and the monkey brain (e.g., Kobatake et al., 1998; Baker et al., 2002).

When tackling the question of how learning influences tolerance for variations in appearance, two general answers have been provided. A first set of theoretical proposals have proposed a role of temporal contiguity, the extent to which different viewpoints of an object have been seen in close temporal proximity. A second possibility is that learning improves tolerance without a need for temporal contiguity.

### Building Tolerance through Temporal Contiguity

Theorists have emphasized the role of temporal contiguity of visual features in linking together multiple images of the same object into one object representation (Földiák, 1991; Rolls, 1992). During natural visual experience, different views of one and the same object are often seen in rapid succession, thus being temporally contiguous. The visual system could take advantage of these natural statistics and learn to associate images that quickly succeed each other to yield tolerant object recognition (Li and DiCarlo, 2008, 2010, 2012).

Behavioral experiments in humans as well as single neuron studies in monkeys have provided evidence consistent with this hypothesis. In most of those studies, subjects are presented with rapid sequences of images that do not correspond to different views of the same object, as such being at odds with the aforementioned natural statistics. If the temporal contiguity hypothesis is correct, the visual system should start to incorrectly associate the views of the different objects that were presented in the same sequence. In the terminology of some of these studies, the typical tolerance for changes in appearance would be broken (“breaking invariance”). Cox et al. (2005) investigated position tolerance in humans. Subjects had to make a saccade toward an object that was presented in the periphery, either to the left or right of the fixation point. Unbeknownst to the subjects, the object identity changed during the saccade in half of the trials, named ‘swap trials.’ The results revealed that, during a same-different task after exposure, subjects more often confused object pairs that had been swapped across retinal positions. This indicates that different objects that were paired in a temporally contiguous way became more likely to be viewed as the same object (Cox et al., 2005).

Another behavioral experiment in humans showed evidence for the temporal contiguity hypothesis for face recognition (Wallis and Bülthoff, 2001). Subjects were shown sequences of faces that rotated in depth, and the identity of the face changed as the head rotated. Discrimination performance in a same-different task after exposure was significantly worse for face pairs that had been paired in a sequence than for face pairs that had never been associated in this way. The same study also suggested that spatiotemporal rather than temporal contiguity is needed for two different faces to become associated and be treated as the same. A recent study by Tian and Grill-Spector (2015) on unsupervised learning of viewpoint tolerance in objects qualified this conclusion by showing that the extent to which spatiotemporal contiguity during training provides an advantage over temporal contiguity alone can vary. When many views are provided during training, temporal proximity of the views is sufficient to improve view-invariant object recognition. When fewer views are shown during training, spatiotemporal contiguity provides benefits to performance and also increases generalization to new views. A study by Wallis et al. (2009) revealed that training with valid as well as invalid face sequences (i.e., sequences of the same face and sequences comprising of two different faces, respectively) either rotating in plane, rotating in depth or varying in illumination led subjects to increasingly assign the images to a single person.

Li and DiCarlo (2008, 2010, 2012) provided evidence for the temporal contiguity hypothesis at the level of single neurons in the inferior temporal cortex (ITC) in macaque monkeys. They compared the effect of training with swap trials and non-swap trials. These studies showed that swapping objects across retinal positions or sizes can reduce and even reverse neurons’ object selectivity. These effects were independent of the size and timing of reward.

### Building Tolerance without Temporal Contiguity

Several studies have suggested that experience with temporal contiguity is not required, at least not for viewpoint tolerance. In a study by Wang et al. (2005), monkeys were trained to discriminate between objects within each of four viewing angles (0, 30, 60, and 90°). This task did not provide the opportunity to associate different views of the same object. Nonetheless, the monkeys were able to discriminate the objects across viewpoint changes up to 60° after the preparatory discrimination task, but not without such preparatory experience. Yamashita et al. (2010) refined this observation by showing that the preparatory task only results in tolerant object recognition if the differences between the discriminated objects are subtle, and that discriminating very different objects is insufficient. The neural substrates of this phenomenon were studied by Okamura et al. (2014). They showed that, after the preparatory discrimination task, neurons in the monkeys’ infero-temporal cortex not only showed object-selective responses at the preferred viewing angle, but also at views 30 and 60° away of the preferred angle. The neural viewpoint invariance was comparable to that after a task that required view-invariant discrimination. No neural viewpoint invariance was observed without the preparatory task. Similarly, a recent study by Soto and Wasserman (2016) showed in pigeons and humans that discrimination training with affine transformations (size, planar rotation, shear) of a single viewpoint of the objects can result in viewpoint-invariant recognition.

These studies thus jointly suggest that temporal contiguity might not be necessary for the emergence of viewpoint tolerant object recognition. Nonetheless, the training phases in these studies were in general much longer than in the temporal contiguity studies in monkeys (multiple weeks compared to a single session) and subjects were consistently rewarded for correct answers. In contrast, the temporal contiguity effects were established in a much shorter timeframe and independent of reward. While the studies mentioned in this paragraph thus indicate a useful alternative learning process, learning through temporal contiguity might be more automatic and more reflective of naturally occurring situations.

### The Present Study on Temporal Contiguity Learning

Notwithstanding the importance of the previous work on temporal contiguity, there are inconsistencies and missing links in the state of the art. In humans, only behavioral studies have been performed, while all monkey studies used single-neuron electrophysiology. Human fMRI studies would thus be an important addition to link the behavioral work to underlying neural mechanisms. This is particularly relevant because there is a clear discrepancy between the two sets of studies. In human behavior, temporal contiguity modulates the discriminability of small object differences, and the overall effect size is relatively small. In monkey electrophysiology, swapping object identity can result in much more massive effects, so that neurons are no longer able to differentiate very different images, say, a boat and a soccer ball.

The current study’s design is based on the human behavioral studies, thus, we want to investigate the behavioral and neural selectivity and tolerance for small object differences, in our case faces. It is a challenge to pick up a neural signature for such small object differences with human functional imaging. They cannot be picked up with conventional fMRI analyses. Even the popular and more sensitive multi-voxel pattern analysis (MVPA, Norman et al., 2006) has its limitations in this respect. In particular, MVPA has shown rather small sensitivity to differences in faces in previous studies (e.g., Kriegeskorte et al., 2007; Nestor et al., 2011; Goesaert and Op de Beeck, 2013), which is in line with its very modest sensitivity for other within-class subordinate object distinctions (e.g., Eger et al., 2008a,b; Brants et al., 2016). An overall lesson seems to be: if objects or faces are so similar that they might be confused by (naïve) human observers, then MVPA would show no or very limited ability to differentiate those stimuli. For that reason, we designed our studies and stimulus sets for a different fMRI methodology, namely fMRI adaptation. FMRI adaptation (fMRIa) can be used to infer selectivity within a neural population, because neural responses tend to be reduced when the same or a highly similar stimulus is repeatedly presented, a principle called Repetition Suppression (RS) (Grill-Spector et al., 2006; Krekelberg et al., 2006). With this method, fine within-category distinctions can be detected (Panis et al., 2008; Gillebert et al., 2009).

FMRI adaptation has already been used to probe face representations. Adaptation in face-selective regions to the identity of faces that are consistently presented in the same viewpoint is a robust finding in the literature (e.g., Grill-Spector and Malach, 2001; Soon et al., 2003; Andrews and Ewbank, 2004; Winston et al., 2004). Studies on adaptation across viewpoint have been much sparser. Studies vary in design and the range of viewpoints that is included, as well as the amount of change faces undergo besides changes in viewpoint (e.g., changes in expression, lighting, etc.), and results are inconclusive. Even for studies using very small changes in viewpoint (i.e., as small as 8°, up to 20°), results vary from finding no adaptation across viewpoints in face-selective regions (Xu et al., 2009), to finding adaptation across viewpoints for identity (FFA, OFA) as well as expression (OFA) for unfamiliar faces (Xu and Biederman, 2010), with another study finding adaptation across viewpoints for familiar faces only (Ewbank and Andrews, 2008). Studies that use large changes in viewpoint do not paint a much clearer picture. One study found partial adaptation across viewpoint in face-selective regions (Pourtois et al., 2005b). Multiple studies reported across-view adaptation effects outside of the face-selective regions (Pourtois et al., 2005a, 2009), sometimes modulated by familiarity (Eger et al., 2005; Pourtois et al., 2005a), while another study did not find any orientation tolerance (Fang et al., 2007).

The current study starts from the mixed evidence on viewpoint tolerance at the neural level, and aims to investigate to what degree training can improve or degrade viewpoint tolerance. The current study consists of a behavioral and an fMRI adaptation experiment. Both experiments investigate the plausibility of the temporal contiguity hypothesis, using a training paradigm with temporally contiguous sequences of faces that either keep or change their identity across changes in viewpoint. As such, the behavioral experiment provides an independent replication of the orientation tolerance experiment of Wallis et al. (2009), while the fMRI experiment aims to further clarify the neural underpinnings of this phenomenon. We expect our results to be in accordance with the temporal contiguity hypothesis. In the behavioral experiment, we thus expect to replicate the results of Wallis et al. (2009). A general prediction for the fMRI experiment is that associating images in a training paradigm will lead to more adaptation. These predictions will be clarified in more detail in the data analysis section.

## Materials and Methods

### Participants

Twenty naïve volunteers (all female; age 18–19), recruited through the online recruitment system of the Faculty of Psychology and Educational Sciences at KU Leuven, participated in the behavioral experiment in exchange for course credit.

A separate set of 21 subjects (eight female; age 18–41, mean 24.3) participated in the fMRI experiment, and were paid for their time. Participants were recruited through the online recruitment system of the Faculty of Psychology and Educational Sciences at KU Leuven, as well as the first author’s social network. All participants filled in an MRI safety questionnaire prior to starting the experiment. One participant was excluded because of excessive head motion during the scan session. Motion parameters for this participant were within pre-set limits (see section “fMRI Adaptation Experiment”) for only two experimental runs. The fMRI study’s final subject sample thus consisted of 20 individuals (seven female, mean age 24.6).

All participants in both experiments had normal or corrected to normal vision, had no history of neurological or psychological problems, and were right-handed. The experiments were approved by the Medical Ethical Committee of the University Hospital of Leuven (UZ Gasthuisberg). All subjects in both experiments gave written informed consent prior to starting the experiment.

### Stimuli

**FIGURE 1:**
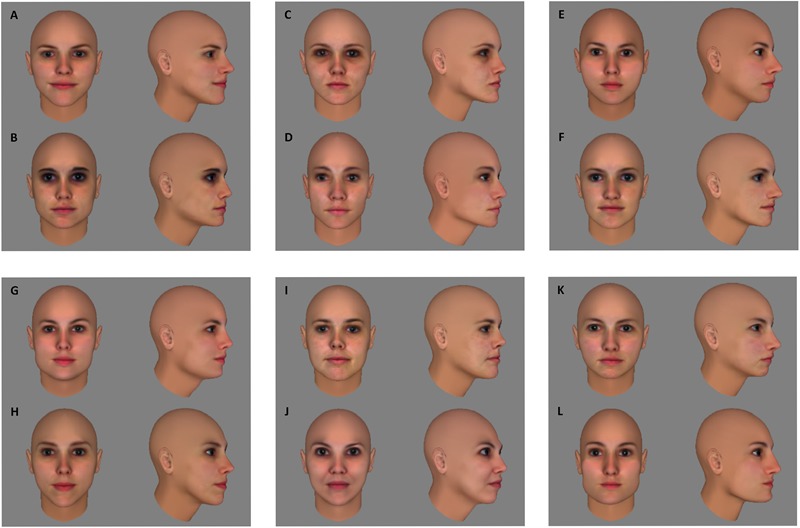
Fifty head models were created in FaceGen Modeller 3.5. The pixel-wise similarity was calculated for all pairs of frontal views and all pairs of 60° views of those 50 head models (detailed description of the similarity index in text). Six face pairs (AB, CD, EF, GH, IJ, KL) that are equivalent in pixel-wise similarity for frontal as well as 60° views were selected from the larger pool to use in the experimental conditions.

The preparation for stimulus construction and selection started with the creation of 50 head models in FaceGen Modeller 3.5 (Singular Inversions), using front and profile pictures of female faces taken from the FEI Face Data Base^1^. For each of the heads, a frontal image (0°) and an image of the head rotated 60° in depth were saved. A pixel-wise (dis)similarity index was calculated for all pairs of frontal views and all pairs of 60° views: the squared difference between the two images’ RGB values was calculated for every pixel and then summed over pixels and over R, G, and B; the final index was the square root of this sum. Six face pairs that are equivalent in pixel-wise similarity for frontal as well as 60° views were selected from the larger pool to use in the experimental conditions (**Figure 1**).

**FIGURE 2:**
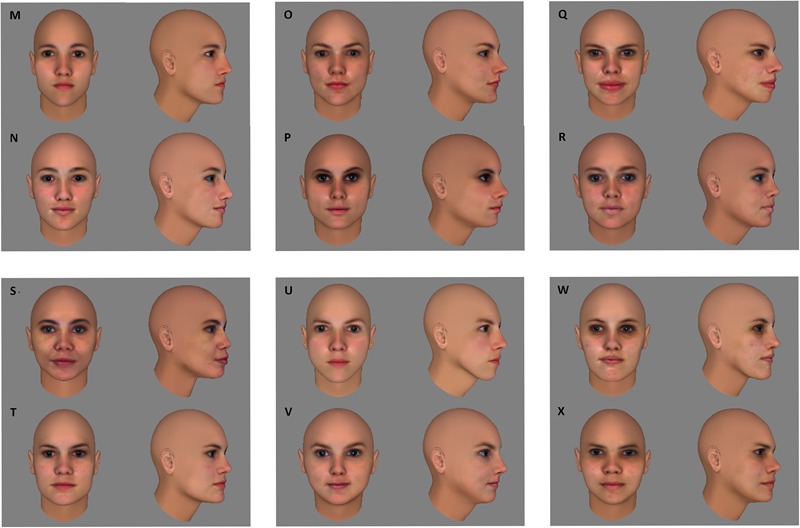
For the control conditions of the fMRI experiment, six extra face pairs (MN, OP, QR, ST, UV, WX) were selected that were equal in pixel-wise similarity to the six face pairs of the experimental conditions (see **Figure 1**).

Before preparing the learning phase, the suitability of this stimulus set was verified in a separate group of ten subjects. We reasoned that without training discrimination performance within face pairs should be above chance and below a perfect score to avoid floor and/or ceiling effects in the learning experiment. The group of ten subjects performed the same/different task that would also be used in the actual experiment (see section “Procedure”). Mean accuracy was 70,75%, with a 95% confidence interval ranging from 66 to 75,5%, thus being significantly different from both chance level [50%; *t*(9) = 9.8735, *p* < 0.0001] and perfect performance [100%; *t*(9) = -13.9181, *p* < 0.0001]. In the fMRI adaptation experiment, this stimulus set was supplemented with six more face pairs for the control conditions, that were equivalent in pixel-based similarity to the face pairs in the experimental conditions (**Figure 2**). An additional six faces – divided in two sets of three – were selected to function as target faces. Each participant was assigned one of the two sets of target faces.

### Imaging Parameters

Data of the fMRI adaptation experiment was collected on a 3T Philips Ingenia CX scanner with a 32-channel coil at the Department of Radiology of KU Leuven. An EPI imaging sequence with TR: 2 s, TE: 30 ms, flip angle 90°, FoV: 210 mm × 210 mm, and matrix size: 104 × 106 was used to obtain functional images. Each volume in the experimental runs consisted of 32 approximately axial slices of 2 mm thick with an in-slice resolution of 2 by 2 mm, and a gap of 0.2 mm. The volumes covered most of the brain, excluding the upper parts of the parietal and frontal lobes as well as a small anterior part of the temporal lobe. For the localizer scans, a TR of 3 s was used, and volumes consisted of 47 approximately axial slices (covering the whole brain). All other scanning parameters stayed the same. Anatomical images were acquired with an MP-RAGE sequence, with a voxel size of 1 × 1 × 1.

### Procedure

The behavioral experiment consisted of a pre-training test phase, a training phase and a post-training test phase. Stimulus presentation was controlled with a Dell desktop (Optiplex 755) running Windows 7 on a 19 inch monitor with a resolution of 1280 × 1024 and a frame rate of 60 Hz. The fMRI adaptation experiment consisted of a training phase outside the scanner, and a post-training scan session comprising eight experimental runs and four localizer runs. Pictures were projected onto a screen and were viewed through a mirror mounted on the head coil. MATLAB (Mathworks, inc.) and the Psychophysics Toolbox 3 (Brainard, 1997) were used to program the stimulus presentation of both experiments. Lights were switched off during the experiments.

#### Training Phase

The training phase was exactly the same for the behavioral and fMRI experiment. During training, participants were presented with 12 different face sequences (2 sequences per face pair). Each sequence consisted of a face being displayed in seven different rotation angles (0–60°, in steps of 10°) at 200 ms per image, with each image being immediately replaced by the following one, creating the appearance of a rotating head. Sequences were played back and forth 3 times before moving on to the following sequence. A fixation cross was presented for 600 ms between sequences. The training lasted approximately 17,5 min in total and consisted of four blocks, separated by 1-min breaks. In each block, all sequences were presented twice in random order.

**FIGURE 3:**
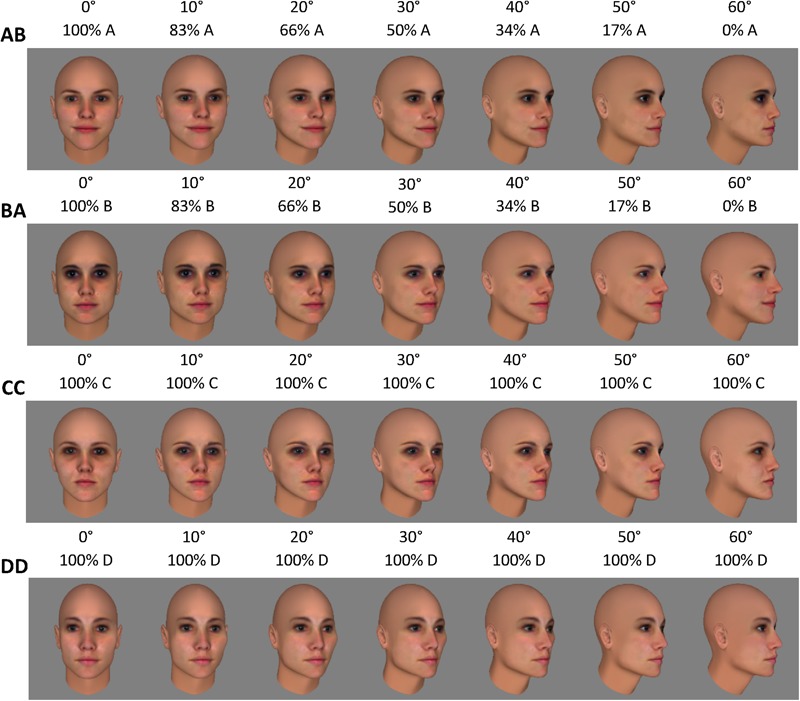
During training, 12 different face sequences (two sequences per pair) were shown. The six experimental face pairs were divided into the morph and non-morph condition. Assignment to both conditions was counterbalanced across participants. This figure presents the sequences for one pair per condition, assuming that pair AB is in the morph condition, and CD is in the non-morph condition. In morph sequences (AB and BA in this figure) the face morphed into the other face of the pair during rotation. In non-morph sequences (CC and DD) faces underwent veridical changes in appearance across the rotation.

For each participant, half of the face pairs were in the “morph condition,” while the other half were in the “non-morph condition.” **Figure 3** provides examples of the two sequences for a face pair in the morph condition and for a face pair in the non-morph condition, respectively. For face pairs in the non-morph condition, the sequences consisted of one face undergoing veridical changes in appearance across the rotation (e.g., sequences CC and DD in **Figure 3**). For face pairs in the morph condition, the sequences were non-veridical: during rotation, the face simultaneously morphed into the other face of the pair (e.g., sequences AB and BA in **Figure 3**). For each face pair, two different sequences were thus shown during training (e.g., training with sequences CC as well as DD; and sequences AB as well as BA). The assignment of face pairs to the two training conditions was counterbalanced across participants. Participants were asked to attentively watch all rotating faces. To safeguard compliance, we stressed the importance of being focused during training when giving verbal instructions to the participants. In the fMRI experiments, we additionally told participants that faces shown during training would reoccur during the fMRI task.

#### Behavioral Experiment: Test Phase

**FIGURE 4:**
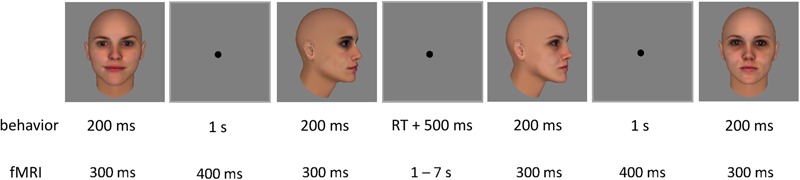
Two example trials of the test phase, with one inter-trial interval. Each trial contained two sequential face images. In the behavioral experiment, each image was presented for 200 ms, with an inter-stimulus interval of 1 s. A fixation cross was shown for 500 ms after participants’ response before the next trial started. In the fMRI adaptation experiment, each image was presented for 300 ms each, with an inter-stimulus interval of 400 ms. The inter-trial interval was jittered between 1 and 7 s.

The test phase of the behavioral experiment consisted of a same-different task. On each trial, participants indicated by button press whether they thought two sequentially presented face images depicted the same or a different identity (**Figure 4**). Each trial started with a fixation cross presented for 500 ms, followed by the two face images – one frontal view (0°), and one side view (60°) – each presented for 200 ms with a 1 s stimulus interval. The two images within a trial always belonged to the same face pair, resulting in four possible combinations: frontal view of face 1 and side view of face 1; frontal view of face 1 and side view of face 2; frontal view of face 2 and side view of face 2; frontal view of face 2 and side view of face 1. All combinations were presented for all pairs, and in both orders (frontal view – side view, and side view – frontal view), resulting in 48 trials per block. Trials can be split up in the following experimental conditions: (1a) ‘morph same’: same identity trials for faces that have been morphed during training; (1b) ‘morph different’: different identity trials for faces that have been morphed during training; (2a) ‘non-morph same’: same identity trials for faces that have not been morphed during training; and (2b) ‘non-morph different’: different identity trials for faces that have not been morphed during training. Responses were self-paced. Participants completed four blocks before training, and four blocks after training. Blocks were separated by 20 s breaks.

#### fMRI Adaptation Experiment: Experimental Runs

During the experimental runs of the fMRI experiment, stimuli were presented in an event-related design fit for fMRI adaptation (**Figure 4**). Each trial contained two sequential face images, presented for 300 ms each, with an inter-stimulus interval of 400 ms. These timing parameters have previously been used in adaptation studies with objects (Kourtzi and Kanwisher, 2000, 2001; Jiang et al., 2007; Dilks et al., 2011) and faces (Winston et al., 2004; Jiang X. et al., 2006; Xu et al., 2009; Xu and Biederman, 2010). The inter-trial interval was jittered between 1 and 7 s to increase the design’s efficiency (optseq2^2^). A fixation cross was presented for 6 s prior to the first trial as well as after the last trial.

The experimental conditions consisted of the same image combinations as those in the behavioral experiment’s test phase: trials contained one frontal view (0°) of a face and one side view (60°) in either order, and images depicted either the same or a different identity for faces that were morphed or not morphed during training.

Aside from these experimental conditions, a number of control conditions were added. In these conditions, images of faces that were not presented during training were used (**Figure 2**). In the ‘same-view condition,’ two frontal views (0°) or two side views (60°) of the same or a different identity (within a face pair) were shown. These trials served to verify whether our design can pick up adaptation under circumstances where we know it to be present (image repetition without any need for viewpoint tolerance). In the ‘untrained-across-view condition,’ one frontal view (0°) and one side view (60°) of the same or a different identity were shown. In other words, these trials were entirely comparable to those of the experimental conditions and only differed from them in that the images contained faces that were not presented during training. The untrained-across-view condition functions as a benchmark: By comparing the amount of adaptation – or the lack thereof – in the experimental conditions to that in the untrained-across-views condition, the directionality of training effects can be revealed (decrease or increase of adaptation across viewpoints). The assignment of face pairs to the same-view and untrained-across-view conditions was counterbalanced across participants.

The task differed from that in the behavioral experiment. During the experimental runs in the scanner, subjects performed a target detection task. They were required to respond by button press when an image (frontal or side view) of either of three target faces was shown. A target face image was always paired with an image of a control face, and could be presented as the first or second image of the trial. This task was chosen for the following reasons: it requires participants to pay attention to each and every image; it is independent of whether or not the two images in a trial depict the same identity, thus allowing for stimulus-driven adaptation effects and minimizing the influence of decision processes on the results. The three target faces were shown to participants after training, just prior to starting the experimental runs. In this preview, the frontal and side view of each target face were presented together for 10 s, this was then repeated, after which all three faces were shown together for 20 s.

Each experimental run consisted of 96 trials (12 per condition), plus 24 target trials, and lasted 6 min 20 s. Experimental runs always preceded the localizer runs.

#### fMRI Adaptation Experiment: Localizer Runs

Face- and object-selective regions (ROIs) were defined using localizer scans. Subjects passively viewed blocks of grayscale pictures of faces, non-living objects and scrambled textures. Each run consisted of 15 blocks of 15 s. The first, middle and last block were fixation blocks. In each of the other blocks, 20 pictures of one category (faces/objects/scrambled) were presented for 300 ms each, with blanks of 450 ms between images. Each category was presented four times, in pseudo-random order with the restriction that each category was presented twice before and twice after the middle fixation block. Participants performed a one-back repetition detection task by pressing a button when the same picture was presented two times in a row. Three repetitions were presented per block.

## Data Analyses

### Behavioral Experiment

The data of the test phases can be split up into four trial types based on whether the two images in a trial depict the same or a different identity and whether they belong to face pairs that were morphed during training or not. **Table 1** clarifies which trial types contain images that were part of the same training sequence and thus associated with each other through training. Gain scores were calculated for all trial types for all participants by subtracting baseline performance (i.e., the performance before training) from post-training performance to exclude potential influence of subtle pre-existing variation between conditions on the results. A repeated-measures ANOVA was performed on the pre-training data as well as on the gain scores, with same/different and associated/not associated as within-subject factors. Paired *t*-tests were performed separately for same identity trials and different identity trials. We expect an interaction effect reflecting an increase in gain scores for same identity trials that were associated during training and/or a decrease in gain scores for different identity trials that were associated during training.

**Table 1:** Test phase trial types and the association of the presented images during training.

	Morphed	Non-morphed
Same identity	Not associated during training	Associated during training
Different identity	Associated during training	Not associated during training

### fMRI Adaptation Experiment

#### Preprocessing and General Linear Model

The Statistical Parametrical Mapping software package (SPM 12, Welcome Department of Cognitive Neurology, London), MATLAB (Mathworks, inc.) and R (R Core Team, Vienna, Austria) were used for preprocessing and analysis of the imaging data. Functional images were slice time corrected (with the first image as reference), and then spatially realigned (to the mean image) to correct for head motion. Motion larger than the size of one voxel (2 mm) between two adjacent time points within a run was defined as excessive, and resulted in the exclusion of that run. This resulted in the exclusion of 1 localizer run for 1 subject, 1 experimental run for another subject, and 3 experimental runs for a third subject. The remaining images were coregistered to the anatomical images, normalized to MNI space (with the SPM 12 European brains template) and spatially smoothed (4 mm FWHM kernel).

The preprocessed data was modeled for each voxel, for each run, and for each participant using a General Linear Model (GLM). The GLM of the experimental runs included eight regressors for the conditions of interest, a regressor for the target trials, and six regressors for the motion correction parameters (*x, y, z* for translation and rotation). False alarm trials – i.e., non-target trials where participants reported having seen a target – were modeled out with a separate regressor (results were very similar without modeling false-alarm trials). Target trials and false alarm trials were not further analyzed. The GLM for the localizer runs included three regressors of interest (for faces, objects and scrambled textures) and six motion regressors.

#### Regions of Interest

We defined subject-specific face-selective and object-selective ROIs using the functional images of the localizer runs. Each subject’s functional activity map was thresholded at *p* = 0.0001 and masked with face and object parcels^3^ of Julian et al. (2012). Activity maps for the face > objects contrast were masked with FFA, OFA, and STS parcels, respectively, to select the face-selective ROIs. Activity maps for the objects > scrambled contrast masked with Julian et al.’s LOC parcel were used to select object-selective LOC. Information about the size and location of these parcels is presented in **Table 2**. LOC was then manually divided into LO and pFs for each subject. ROIs needed to contain at least 20 active voxels within either hemisphere to be selected. If both hemispheres showed activity in less than 20 voxels for a particular ROI, the threshold was lowered for that ROI until at least 20 voxels (within a hemisphere) could be selected. This resulted in a more lenient threshold in at most 30% of the subjects per ROI (FFA: *p* = 0.01 for 1 subject; OFA: *p* = 0.001 for 1 subject, and *p* = 0.01 for three subjects; STS: *p* = 0.001 for two subjects, and *p* = 0.01 for four subjects). For one subject, we failed to find OFA even at a threshold as lenient as *p* = 0.01. This procedure for adapting the threshold is similar to what has been done before in Goesaert and Op de Beeck (2013). Note that the parcels of Julian et al. (2012) were created using an unchanged threshold of *p* = 0.0001 and robustness to changes in threshold was not tested. In case of overlap between face-selective and object-selective ROIs, the face-selective voxels were excluded from the object-selective ROIs. The object-selective regions were selected as control regions.

**Table 2:** Size and location information for the parcels used to define the regions of interest [information taken from Julian et al. (2012), their **Table 1**], and size information for the ROIs in our study.

	Parcel size (mm^3^)	Parcel peak MNI coordinates	Average ROI size (mm^3^)
Left FFA	4248	-40 -52 -18	472 (*n* = 16)
Right FFA	8152	38 -42 -22	926 (*n* = 20)
Left OFA	1688	-40 -76 -18	352 (*n* = 8)
Right OFA	6320	44 -76 -12	675 (*n* = 18)
Left STS	6752	-54 -38 6	299 (*n* = 3)
Right STS	20040	48 -38 4	594 (*n* = 20)
Left LOC	39768	-46 -72 -4	7431 (*n* = 20)
Right LOC	40680	46 -70 -4	7503 (*n* = 20)

#### Adaptation Analysis

The functional images of the experimental runs were used to perform the adaptation analysis. Beta values were extracted for each condition, for each participant, and averaged across voxels within each ROI. Beta values were then averaged across right and left hemispheres for each ROI (e.g., beta values for rFFA and lFFA were averaged into one beta value for FFA) for each participant. For each condition and each ROI, the mean beta value across participants was then computed. We did not formally test for lateralization of the effects, because it regularly happened that face-selective ROIs existed in only one of the two hemispheres, leaving relatively few data to make the comparison between hemispheres. Face-selective ROIs were more often missing from the left compared to the right hemisphere and, when found, were smaller than those in the right hemisphere.

Adaptation to identity can be revealed by comparing beta values of same-identity trials to beta values of different-identity trials of the same condition: Given that neural activity decreases when the visual system adapts to an image, lower beta values for same-identity trials compared to different-identity trials are evidence for adaptation to the identity of the faces. As our hypotheses are directional and specific to conditions, we used *a priori* one-tailed paired *t*-tests. In the same-view condition, we expected beta values of same-identity trials to be significantly lower than those of different-identity trials. As same-image adaptation has been reported extensively in the literature (e.g., Grill-Spector and Malach, 2001; Soon et al., 2003; Andrews and Ewbank, 2004; Winston et al., 2004), not finding an adaptation effect in the same-view condition would raise questions about the study’s power to measure adaptation. The presence or absence of an adaptation effect in the other control condition, the untrained-across-view condition, determines which effect is to be expected in the morph and non-morph conditions of interest. If adaptation is found for untrained faces across views, we expect this effect to decrease or disappear in the morph condition (cf. “breaking invariance”), and/or increase in the non-morph condition. If adaptation to identity is absent in the untrained-across-view-condition, we expect it to arise in the non-morph condition, and/or reverse in the morph condition.

## Results

### Behavioral Experiment

Overall performance before training was at 73.1%. As expected, the repeated-measures ANOVA on the pre-training test data revealed no significant effects [same/different: *F*(1,19) = 0, *p* = 0.987; associated/not associated: *F*(1,19) = 1.242, *p* = 0.279; interaction: *F*(1,19) = 0.319, *p* = 0.579].

The repeated-measures ANOVA on the gain scores revealed no effect of whether the two images in a trial depicted the same or a different individual [*F*(1,19) = 2.957, *p* = 0.102] or whether the images had been associated during training [*F*(1,19) = 3.990, *p* = 0.060]. It did, however, reveal a significant interaction [*F*(1,19) = 5.754, *p* = 0.027].

**FIGURE 5:**
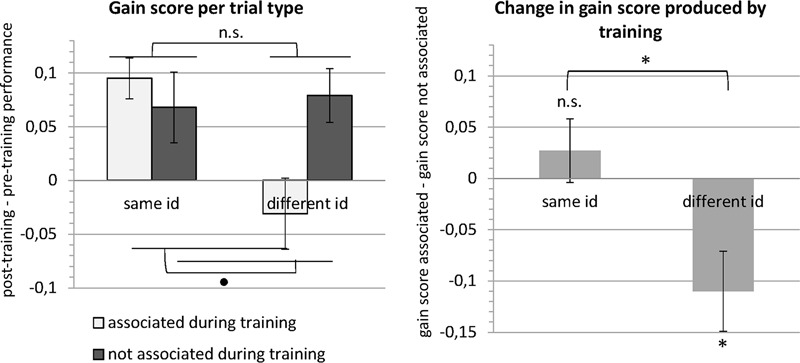
Results of the behavioral experiment. The left panel shows the mean gain scores (i.e., post-training performance – pre-training performance) for the four different trial types (see **Table 1**). A repeated-measures ANOVA revealed that gain scores did not differ between trials with images that depicted the same versus a different identity or between trials with images that were versus were not associated during training. Importantly, there was a significant interaction. In the right panel, the data are expressed in terms of change in gain scores between images that were associated during training versus images that were not associated during training. This change in gain score was significantly different from zero for different identity trials. The negative change for different identity trials means that the gain was lower (images were less well discriminated) for images that were associated during training. Error bars denote the standard error of the mean across subjects (SEM). ^∗^*p* < 0.05, ∙*p* < 0.1, n.s., *p* > 0.1.

When we analyze this interaction further, first for trials with images of the same identity, gain scores did not differ significantly between trials with images that had been associated (mean: 9.5%) and trials with images that had not been associated during training [mean: 6.8%; *t*(19) = 0.8661, *p* = 0.397, paired *t*-test]. Gain scores on trials with images of different identities, however, differed significantly between trials with associated images (mean: -3.1%) and trials with non-associated images [mean: 7.9%; *t*(19) = -2.818, *p* = 0.011, paired *t*-test]. These results suggest a selective drop in performance for trials showing different faces that had been associated during the training through morphing, indicating that these faces were more often confused, with performance even dropping below pre-training levels (evidenced by the negative gain score). These results are summarized in **Figure 5**.

### fMRI Adaptation Experiment

The mean sensitivity index *d*′ (*Z* score of the hit rate – *Z* score of the false alarm rate) for the target detection task was 1.54, indicating that participants paid attention to the faces.

FFA, STS, LO, and pFs were selected in all subjects. In one subject, we did not find OFA in either hemisphere.

**FIGURE 6:**
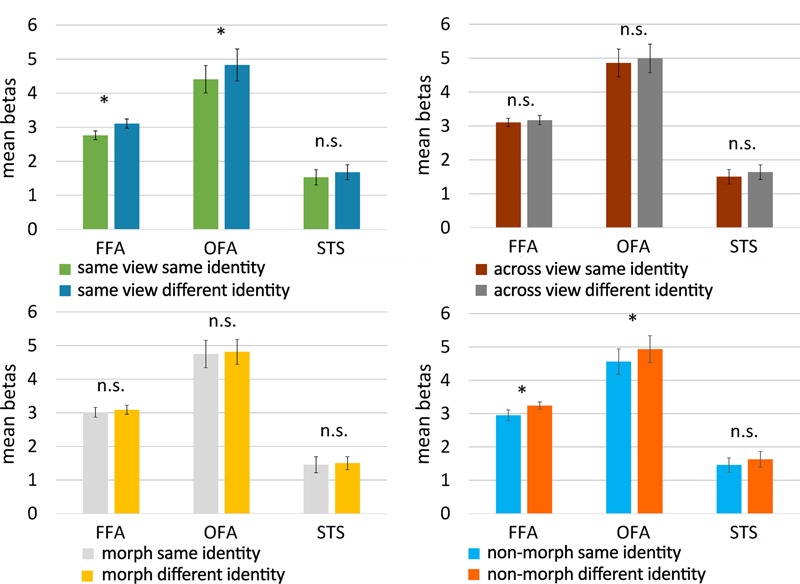
Results of the fMRI adaptation experiment. Bar plots represent mean beta values (extracted from the GLM) per condition and per face-selective ROI. Beta values were averaged across hemisphere for every participant and every ROI before computing the mean. The upper panels show the results for the control conditions (untrained faces), the lower ones present the results for the experimental conditions (morphed and non-morphed faces). Because of the directional hypotheses in the control conditions and non-morph condition, one-tailed *t*-tests were used there. For untrained faces, FFA and OFA adapted to the identity of faces when they were shown in the same view, but not when across-view images were shown. Across-view adaptation to identity was not found for faces that were morphed during training, but did emerge in FFA and OFA for faces that were not morphed during training. Error bars denote the standard error of the mean across subjects (SEM). ^∗^*p* < 0.05; n.s., *p* > 0.05.

Results for the face-selective ROIs are summarized in **Figure 6**. In the same-view control conditions, adaptation to identity was found in FFA [*t*(19) = -2.410, *p* = 0.013, one-tailed paired *t*-test] and in OFA [*t*(18) = -1.869, *p* = 0.039, one-tailed paired *t*-test], but not in STS [*t*(19) = -1.281, *p* = 0.108, one-tailed paired *t*-test; see **Figure 6**, upper left panel]. Further control analyses also found an adaptation effect in the object-selective ROI LO [*t*(19) = -1.839, *p* = 0.041, one-tailed paired *t*-test], but not in pFs [*t*(19) = -1.640, *p* = 0.059, one-tailed paired *t*-test]. Our design is thus able to pick up adaptation effects, at least in FFA, OFA, and LO.

Given the findings with the same-view conditions, the face-selective ROIs which would merit further attention would be FFA and OFA. In the following we continue to provide the statistics of all ROIs for completeness. FFA and OFA did not show an adaptation to identity across views for untrained faces [FFA: *t*(19) = -0.482, *p* = 0.318; OFA: *t*(18) = -0.712, *p* = 0.243, one-tailed paired *t*-tests; see **Figure 6**, upper right panel], nor did any of the other ROIs [STS: *t*(19) = -0.818, *p* = 0.212, LO: *t*(19) = 0.179, *p* = 0.930; pFs: *t*(19) = -1.223, *p* = 0.118, one-tailed paired *t*-tests]. This is an important benchmark. Indeed, given these results, we can refine our predictions for the morph and non-morph conditions as explained in the data analysis section. In the non-morph condition, same-identity trials depict images that have been associated extensively during training, while images of different identities have never been seen together during training. If the training causes neural learning in the non-morph condition, across-view tolerance will arise and reveal itself in an adaptation effect. In the morph condition, however, images of different identities have been associated in a controlled way while same-identity images have not been seen together during training. As this procedure disrupts the “natural” learning process that is simulated in the non-morph condition, we definitely do not expect a normal adaptation effect in the morph condition. If anything, a reverse adaptation effect – with lowered activity for different identity trials compared to same identity trials – could occur as a result of the association procedure.

In accordance with these predictions, we found evidence for adaptation to identity in the non-morph condition in FFA [*t*(19) = -1.782, *p* = 0.045, one-tailed paired *t*-test], as well as in OFA [*t*(18) = -2.020, *p* = 0.029, one-tailed paired *t*-test; see **Figure 6**, lower right panel], but not in the other ROIs [STS: *t*(19) = -0.675, *p* = 0.254; LO: *t*(19) = -0.968, *p* = 0.173; pFs: *t*(19) = -0.960, *p* = 0.174, one-tailed paired *t*-tests].

However, in the morph condition, we found no evidence for a difference in beta values between same-identity and different-identity trials in the face-selective ROIs [FFA: *t*(19) = -0.609, *p* = 0.550; OFA: *t*(18) = -0.463, *p* = 0.649; STS: *t*(93) = -0.373, *p* = 0.714, two-tailed paired *t*-tests; see **Figure 6**, lower left panel] or the object-selective ROIs [LO: *t*(19) = -0.097, *p* = 0.924; pFs: *t*(19) = -1.585, *p* = 0.129, two-tailed paired *t*-tests].

The results thus suggest that adaptation to the identity of new faces is not tolerant for viewpoint, nor is it when identities were mixed up during training. The only condition showing viewpoint tolerance was the training condition in which one and the same identity was shown across viewpoint changes. Note, however, that our tests were uncorrected for multiple comparisons, and that the results of the experimental conditions would not survive Bonferroni correction, indicating that these effects are relatively weak.

## Discussion

In two separate experiments, a behavioral and an fMRI adaptation experiment, we provided evidence in favor of the temporal contiguity hypothesis. In the behavioral experiment, temporal contiguity training produced a specific drop in performance for trained non-veridical view pairings, suggesting that these faces are more often incorrectly judged to be one and the same face. In the fMRI experiment, there was no evidence for viewpoint tolerance without training. However, adaptation across viewpoints arises after training with veridical, non-morphed face sequences, suggesting that temporal contiguity can indeed play a role in generating viewpoint tolerance.

Our behavioral results show the same pattern as the results of the depth rotation experiment of Wallis et al. (2009), but are less pronounced. We replicated the interaction effect, and found performance in trials with different faces to be impaired by non-veridical training, but our effects are notably smaller and we did not replicate the improvement veridical training provided for trials with same face images. A difference in design between their study and ours might partly explain the discrepancy in results. While Wallis et al. (2009) tested subjects after training only – in multiple training-then-test blocks –, we decided to include a pre-training test. Our subjects might thus have been more familiar with the faces before training, which might have reduced the possibility to induce change in performance by training. General performance also seems to have been notably higher in our study. The sparsely available information about performance scores in Wallis et al. (2009) suggests that performance in the depth rotation experiment for views that were not associated during training is around 65%, while it amounts to just above 79% in our experiment. This already high performance might leave less room for additional improvement through temporal association. Our results are very similar to those of the planar rotation experiment of Wallis et al. (2009), both in pattern and effect sizes, but we have no information about general performance in that experiment.

The small effect sizes are not limited to the behavioral study. The effects found in our fMRI study are weak as well. Support for the temporal contiguity hypothesis lies in the combination of an adaptation effect in the non-morph condition in FFA and OFA and the lack thereof in the morph condition. However, it should be noted that while a lack of adaptation in the morph condition is not inconsistent with the temporal contiguity hypothesis, a reversed adaptation effect (with lower activation for the associated different-identity images compared to non-associated same-identity images) would provide stronger evidence in favor of the hypothesis. As noted before in the Section “Results,” the effects reported here are uncorrected for multiple comparisons, and would not survive corrections for all tests that are performed. Moreover, we performed a complementary ANOVA for the experimental conditions, with morph/non-morph and same/different as factors. This ANOVA did not result in a significant interaction in any of our ROIs, highlighting once again that the reported effects are small. Thus, even though the results of this first human imaging experiment trying to demonstrate effects of temporal contiguity are at least compatible with the predictions, the amount of evidence is still weak. Replication studies are needed to evaluate the robustness of the neural training effects, and might benefit from a longer training phase and a higher number of subjects to increase statistical power.

While the behavioral experiment and the neuroimaging experiment both provide findings that are in accordance with the predictions of the temporal contiguity hypothesis, there is a clear discrepancy in results. In the behavioral data, there is evidence that non-veridical training decreases tolerance (i.e., “breaks invariance”), but no evidence for an increase in tolerance after veridical training. In the fMRI data, we see the opposite pattern of results: veridical training resulted in increased tolerance, but no change was seen after non-veridical training. We think that this discrepancy might, at least partially, be a consequence of the difference in baseline viewpoint invariance in the two data sets. In the behavioral experiment, pre-training discrimination performance across views was already high (73.1% correct), indicating a non-negligible extent of viewpoint tolerance. On the other hand, viewpoint tolerance was absent in the fMRI data for untrained faces. It is possible that the emergence of new correct tolerance is a more likely outcome than the expansion of already existing tolerance, and that breaking existing invariance is more likely than reversing selectivity to build new incorrect invariance. Li and DiCarlo (2010) provide results in favor of this idea. In their study, veridical training led to significant increases in tolerance in units with low initial size/position tolerance, but not in units with high initial tolerance. Conversely, non-veridical training resulted in significant decreases only in units with high initial tolerance (see their Figures 6, 7). The larger invariance in behavioral compared to neural data is in itself an interesting point to note, and could be explained. First, there might be an invariant neural representation that we fail to pick up with fMRI. Second, the amount of invariance in behavioral performance could result from computational steps applied during read-out of the less invariant neural representations.

Our analyses focused on face-selective ROIs that were selected using a standard localizer with static images of faces, objects and scrambled objects. A recent study by Guntupalli et al. (2017) suggests that a more anterior face-selective region exists that plays a role in view-invariant representations of identity. This inferior frontal face area (IFFA) has been identified previously using more dynamic face stimuli (e.g., Duchaine and Yovel, 2015), but seems difficult to identify using static images. Guntupalli et al. (2017) used MVPA to decode identity and head view and found a cluster in rIFFA that classified identity across head views. Interestingly, their results suggest that representations in FFA constitute an intermediate stage, as both identity and head view could be classified accurately in FFA. In OFA, only classification of head view, and not identity, was successful. This indication that OFA and FFA might combine view-dependent and view-invariant representations could provide an additional partial explanation for our small effects in these regions.

More in general, other properties of the stimuli that we included might be relevant to consider as well. Our decision to use faces and viewpoint changes was motivated by the fact that much of the behavioral evidence for effects of temporal contiguity was obtained with these stimuli (Wallis and Bülthoff, 2001; Wallis et al., 2009). However, not all faces are the same. The face recognition literature is characterized by a large variety in stimuli: natural photos or computer rendered images, presented in color or grayscale, cropped to exclude external information such as hair or not, etc. It is unknown whether and to what extent these factors would influence effects of temporal contiguity. Nonetheless, arguments can be made to justify our choice of stimuli for this study. Our face stimuli are hairless, thus resembling cropped images. Validated face recognition tests such as the Cambridge Face Memory Test (CFMT; Duchaine and Nakayama, 2006) crop images to exclude non-face information such as hair and parts of clothing, as it has been shown that prosopagnosia patients can still perform within normal ranges when test images include non-face information. Even though natural photos of faces are no doubt the most representative of the faces we encounter in our everyday lives, we used computer-rendered face images to have better stimulus control while still retaining a lot of the properties of naturalistic faces (e.g., skin texture). Studies have indicated that both shape and texture/surface information are important for face recognition (O’Toole et al., 1999; Jiang F. et al., 2006; Caharel et al., 2009; Itz et al., 2017) and both types of information have been suggested to contribute to the holistic processing of faces (Meinhardt-Injac et al., 2013; Zhao et al., 2016). Likely, the performance with our stimulus set is also influenced by shape as well as texture. It could be argued that the inclusion of texture information makes specific faces more easily identifiable across viewpoints, given that certain texture features (e.g., skin color, freckles) might be more viewpoint-invariant than shape features.

A remarkable finding in our study was that in our same-view control condition, we did not only find an adaptation effect in FFA and OFA, but also in LO, one of our control regions. Face identity adaptation effects outside of face-selective regions have been reported in previous studies (Eger et al., 2005; Pourtois et al., 2005a, 2009; Mur et al., 2010). Mur et al. (2010) found adaptation effects in regions that are unlikely to hold face representations (e.g., PPA and early visual cortex), and tried to provide alternative explanations for the effects, which might also be relevant here. Even when tasks do not require participants to attend to differences between faces, a change in face identity might automatically capture attention, and such an attentional response might activate a wider network of regions. Activity in face-selective as well as object-selective cortex has been shown to be influenced by attention (Wojciulik et al., 1998; O’Craven et al., 1999; Murray and Wojciulik, 2004). Other alternative explanations included carryover of activity and responsiveness to stimulus change rather than a stimulus property. It needs to be noted that we only found an adaptation effect in LO in the same-view condition, and that this effect would not survive correction for multiple comparisons. Mur et al. (2010) found that adaptation effects were clearly more widespread in their exact-image repetition condition than in different-image repetition conditions. When exact-image repetitions are used, change in identity and change in stimulus are confounded. This might contribute to the more widespread effects for exact-image repetition, and thus also our same-view condition, but cannot fully explain adaptation effects outside of the face system, as they still found adaptation effects outside of the face system with different-image repetitions, although less widespread. While these alternative explanations call for caution when interpreting the data in terms of neural representation of face identity, it is not impossible that LO contains neurons that respond to the identity of faces, especially considering the fact that this ROI was defined through an object – scrambled contrast.

## Conclusion

In a behavioral experiment and an fMRI adaptation experiment, participants were trained with temporally contiguous veridical (non-morphed) and non-veridical (morphed) face sequences. In the behavioral experiment, morphing faces during training caused a drop in performance for trials with different identities, indicating that the morphing caused the visual system to mix up the morphed identities. In the fMRI experiment, viewpoint tolerance could only be detected after training with non-morphed veridical face sequences, suggesting that exposure training in which images of the same identity are shown across different viewpoints is helpful for viewpoint tolerance. Note, however, that the effect size in the behavioral experiment is smaller than in earlier experiments, and the amount of evidence in the fMRI experiment is also weak from a statistical point of view. The appropriate conclusion is to say that our results are not inconsistent with a role for temporal contiguity, but further empirical testing will be very important.

## Author Contributions

CVM: Paradigm development, stimulus construction, programming of scripts for the experiment, recruitment of participants, data collection, data analysis and writing. HPOdB: Paradigm development, interpretation of data and writing.

## Conflict of Interest Statement

The authors declare that the research was conducted in the absence of any commercial or financial relationships that could be construed as a potential conflict of interest. The reviewer RKO and handling Editor declared their shared affiliation, and the handling Editor states that the process nevertheless met the standards of a fair and objective review.

## Footnotes

1 http://fei.edu.br/~cet/facedatabase.html

2 https://surfer.nmr.mgh.harvard.edu/optseq

3 http://web.mit.edu/bcs/nklab/GSS.shtml
